# A surprising new route to 4-nitro-3-phenylisoxazole

**DOI:** 10.3762/bjoc.6.68

**Published:** 2010-06-23

**Authors:** Henning Hopf, Aboul-fetouh E Mourad, Peter G Jones

**Affiliations:** 1 Institut für Organische Chemie, Technische Universität Braunschweig, Postfach 3329, D-38023 Braunschweig, Germany; 2 Chemistry Department, Faculty of Science, Minia University, 61519 El-Minia, Egypt; 3 Institut für Anorganische und Analytische Chemie, Technische Universität Braunschweig, Postfach 3329, D-38023 Braunschweig, Germany

**Keywords:** cinnamyl alcohol, furoxan derivatives, isoxazole derivatives, X-ray diffraction

## Abstract

A one-pot synthesis of 4-nitro-3-phenylisoxazole has been carried out by treatment of cinnamyl alcohol dissolved in acetic acid with sodium nitrite; in addition, 4-phenyl-3-furoxanmethanol was obtained in 40% yield.

## Introduction

The isoxazole ring system, which can be easily obtained by [3 + 2] cycloaddition of nitric oxides to alkynes, is of interest since it forms a part of various biodynamic agents. Isoxazole derivatives that act as antithrombotic, hypolipidemic, nootropic, antiviral, antiobesity, and CNS modulation agents have been described [1].

On the other hand, derivatives having liquid crystalline properties have received a great deal of attention as they have a wide variety of applications, especially in flat-panel displays [2], light emitting diodes [3–5], anisotropic networks [6–7], and semiconductor materials [8]. Incorporation of the isoxazole moiety into such materials can result in significant changes in the corresponding mesogenic phases, since isoxazoles display classical nematic and smetic mesophases associated with their rod-like core structure [9].

**Scheme 1:**
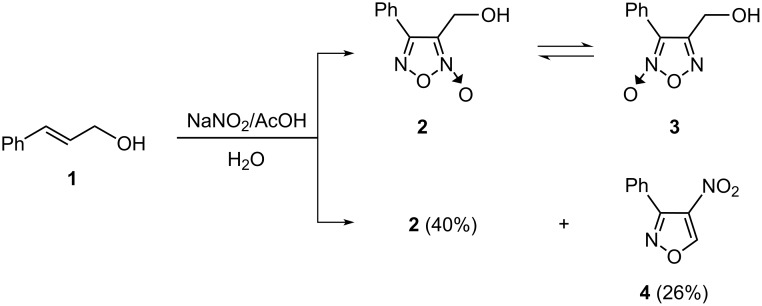
Preparation of **2** and **4** by treatment of cinnamyl alcohol (**1**).

The pioneering work on furoxans as nitric oxide donors by Gasco et al. has stimulated a large number of further studies. One of these reports deals with a synthetic route and structural characterization of two isomeric phenylfuroxans **2** and **3** [10]. According to the original report, 4-phenyl-3-furoxanmethanol (**2**) was obtained as the sole product in 40% yield, by treatment of cinnamyl alcohol (**1**) dissolved in acetic acid with concentrated aqueous sodium nitrite at 70 °C (Scheme 1).

## Results and Discussion

**Figure 1:**
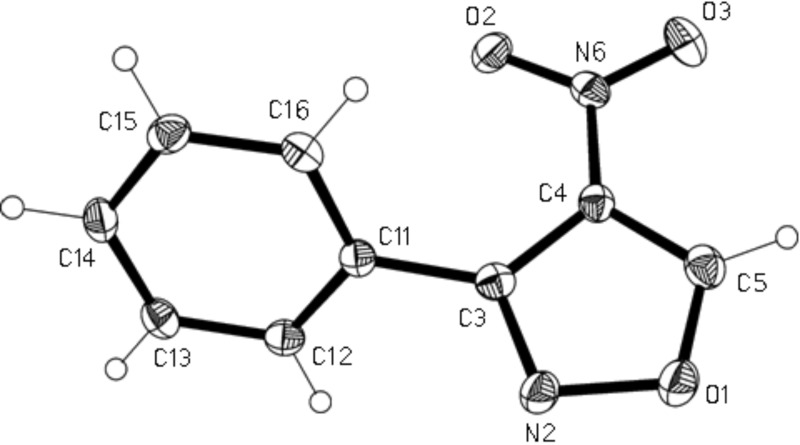
The crystal structure of compound **4**. Ellipsoids correspond to 50% probability levels.

**Figure 2:**
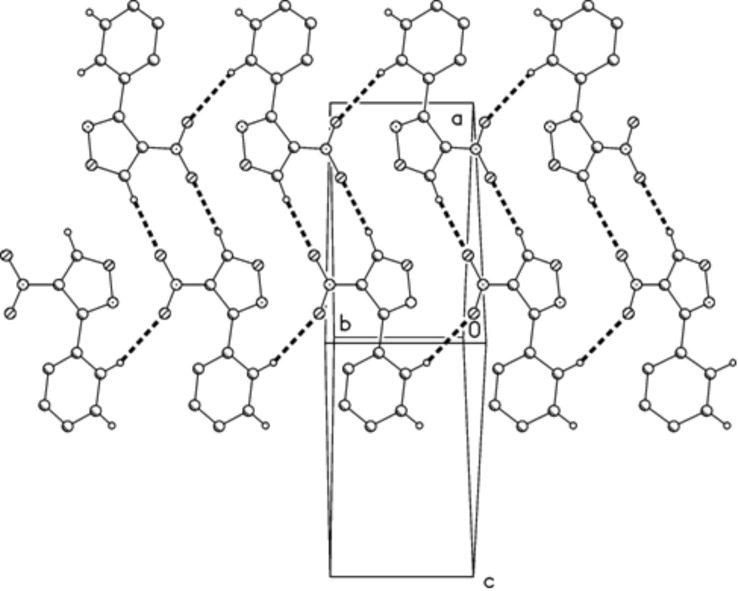
Packing diagram of compound **4** viewed perpendicular to (101). Hydrogen bonds are indicated by thick dashed bonds.

Application of this approach to the synthesis of **2** and purification of the reaction product, as reported, by column chromatography with petroleum ether/ethyl acetate (4:1) as eluant resulted, in our hands, not only in the isolation of the desired reaction product **2** (40%), but surprisingly, also of compound **4** (26% yield). Compound **4** (*R*_f_ = 0.65) was eluted before compound **2** (*R*_f_ = 0.41). The structure of compound **4** was elucidated on the basis of its spectral (see Experimental section) and crystallographic data. The crystal structure of compound **4** is shown in Figure 1. Bond lengths and angles (e.g. C3–N2 1.3110(15), N2–O1 1.4283(13), O1–C5 1.2202(15) Å) may be considered normal. The five-membered ring is planar within a mean deviation of 0.002 Å, and subtends interplanar angles of 6.5° with the nitro and (in the same sense) 58.4° with the phenyl substituent. Molecules are connected to form broad ribbons in the (101) plane and parallel to the short *y* axis (Figure 2) by “weak” hydrogen bonds H12···O2 2.56 and H5···O3 2.39 Å.

**Scheme 2:**
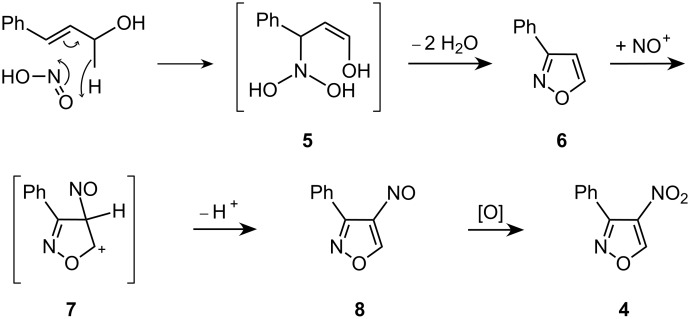
Suggested mechanism for the formation of **4**.

A suggested mechanism for the formation of **4** is outlined in Scheme 2.

We propose that the nitro compound **4** is generated via the intermediate **6**. This, in turn, could be generated by dehydration of the precursor **5**, an addition product of HNO_2_ to the substrate **1** by a formal hetero Alder-ene reaction. Isoxazole **6** could then undergo electrophilic substitution via **7** to afford the nitroso compound **8**, which in a final step would be oxidized to **4** under the reaction conditions.

Previously reported approaches to **4** include the reaction of aromatic α,β-unsaturated aldehydes with dinitrogen trioxide [11–12], the nitration of 3-phenyl-isoxazole [13–14], and the hydrolysis of methyl 4-nitro-3-phenyl-5-carboxylate, obtained by reaction of α-nitroacetophenone oxime and methoxalyl chloride [15].

In conclusion, a very simple route to 4-nitro-3-phenylisoxazole (**4**), as well as the furoxan derivative **2**, which constitutes a nitric oxide (NO) donor site in NO donor systems [16], is disclosed.

## Experimental

Melting points were measured on Büchi 350 apparatus and are uncorrected. IR spectra were measured with Bruker Tensor 27 instrument. ^1^H and ^13^C NMR spectra were recorded in CDCl_3_ with a Bruker DRX-400 instrument operating at 400 MHz and 100 MHz, respectively. Silica gel 60 (Merck, 70–230 mesh) was used for column chromatography. Macherey-Nagel polygram SilG/UV 254 was used for TLC. Petroleum ether refers to the fraction of bp 40–60 °C. The mass spectrum was taken on a Finnigan MAT 4515 spectrometer at 70 eV.

**Preparation of 4-phenyl-3-furoxanmethanol (2) and 4-nitro-3-phenylisoxazole (4):** To a stirred solution of cinnamyl alcohol (**1**, 4.08 g, 30 mmol) in glacial acetic acid (6 mL), was added saturated aqueous sodium nitrite (90 mmol) solution dropwise so that the temperature did not exceed 70 °C. Stirring was continued at room temperature for 1 h. The reaction mixture was diluted with water, extracted with diethyl ether, washed with brine, dried with anhydrous sodium sulfate and concentrated in vacuo. The residue was purified by column chromatography using petroleum ether/ethyl acetate (4:1) as eluent to give 4-phenyl-3-furoxanmethanol (2.29 g, 40%) and 4-nitro-3-phenylisoxazole (1.49 g, 26%), respectively.

**4-Phenyl-3-furoxanmethanol (2):** mp 67 °C (benzene/petroleum ether); lit. mp 66–67 °C [10]; IR: ν = 3127, 1569, 1523, 1449, 1404, 1372, 1195, 1130, 883, 767, 693 cm^−1^; ^1^H NMR: δ = 7.85–7.78 (m, 2H, Ar-H), 7.61–7.58 (m, 3H, Ar-H), 4.75 (s, 2H, CH_2_), 2.75 (br. s, 1H, OH); MS (EI): *m*/*z* = 191 [M]^+^ (42), 174 (7), 145 (12), 129 (16), 103 (100), 93 (8), 76 (38).

**4-Nitro-3-phenylisoxazole (4):** mp 113–114 °C (ethyl acetate); lit. mp 114.5 °C [17]; ^1^H NMR: δ = 9.36 (s, 1H, H-5), 7.70–7.67 (m, 2H, Ar-H), 7.59–7.49 (m, 3H, Ar-H); ^13^C NMR: δ = 160.6 (C-5), 156.5 (C-3), 133.9 (C-4), 131.1, 129.5, 128.6, 124.7 (Ph).

**X-Ray structure determination of 4:** Crystal data: C_9_H_6_N_2_O_3_, *M*_r_ = 190.16, monoclinic, *P*2_1_/*c*, T = −173 °C, a = 12.1621(7), b = 5.6566(3), c = 12.0633(8) Å, β = 101.638(6)°, V = 812.85 Å^3^, Z = 4, F(000) = 392, λ(Mo Kα) = 0.71073 Å, μ = 0.12 mm^−1^, D_x_ = 1.554 g cm^−3^. Data collection: A colourless prism ca. 0.3 × 0.15 × 0.1 mm was mounted in inert oil on a glass fibre and transferred to the cold gas stream of an Oxford Diffraction Xcalibur E diffractometer. A total of 13,352 reflections were recorded to 2Θ 57.2°, of which 2100 were independent (*R*_int_ 0.037). Structure refinement: The structure was refined using SHELXL-97 [18]. Hydrogen atoms were included using a riding model. The final *R*2 (all reflections) was 0.084 for all intensities and 127 parameters, with *R*1 (I>2σ(I)) 0.035; S 0.94, max. Δρ 0.25 e Å^−3^.

X-ray crystallographic data (excluding structure factors) were deposited under the number CCDC-763982 and can be obtained free of charge from the Cambridge Crystallographic Data Centre via http://www.ccdc.cam.ac.uk/data_request/cif.
